# Supplementary material for: Cranial and mandibular shape variation in the genus Carollia (Mammalia: Chiroptera) from Colombia: biogeographic patterns and morphological modularity
Source: PeerJ. 2015 Aug 13;3:e1197. doi: 10.7717/peerj.1197 (PMC4581772; doi:10.7717/peerj.1197)
Supplement: Table S1 — Collection information of specimens of Carollia brevicauda, Carollia castanea and Carollia perspicillata used for this study. [file peerj-03-1197-s001.doc]

**Table A1.** Collection information of specimens of *Carollia brevicauda*, *Carollia castanea* and *Carollia perspicillata* used for this study.

| Number | Species | Code | Institution |
| --- | --- | --- | --- |
| 1 | *C. brevicauda* | MUJ273 | MPUJ |
| 2 | *C. brevicauda* | MUJ287 | MPUJ |
| 3 | *C. brevicauda* | MUJ1203 | MPUJ |
| 4 | *C. brevicauda* | MUJ1537 | MPUJ |
| 5 | *C. brevicauda* | MUJ1592 | MPUJ |
| 6 | *C. brevicauda* | MUJ1545 | MPUJ |
| 7 | *C. brevicauda* | MUJ1618 | MPUJ |
| 8 | *C. brevicauda* | MUJ1623 | MPUJ |
| 9 | *C. brevicauda* | MUJ1621 | MPUJ |
| 10 | *C. brevicauda* | MUJ1206 | MPUJ |
| 11 | *C. brevicauda* | MUJ1120 | MPUJ |
| 12 | *C. brevicauda* | MUJ1101 | MPUJ |
| 13 | *C. brevicauda* | MUA10192 | CTUA |
| 14 | *C. brevicauda* | MUA10471 | CTUA |
| 15 | *C. brevicauda* | MUA10552 | CTUA |
| 16 | *C. brevicauda* | MUA10202 | CTUA |
| 17 | *C. brevicauda* | MUA11233 | CTUA |
| 18 | *C. brevicauda* | MUA10468 | CTUA |
| 19 | *C. brevicauda* | MUA11236 | CTUA |
| 20 | *C. brevicauda* | MUA10525 | CTUA |
| 21 | *C. brevicauda* | MUJ1466 | MPUJ |
| 22 | *C. brevicauda* | MUJ1300 | MPUJ |
| 23 | *C. brevicauda* | MUJ1486 | MPUJ |
| 24 | *C. brevicauda* | IAvH-M-2359 | IAvH-M |
| 25 | *C. brevicauda* | IAvH-M-3358 | IAvH-M |
| 26 | *C. brevicauda* | IAvH-M-3351 | IAvH-M |
| 27 | *C. brevicauda* | IAvH-M-7197 | IAvH-M |
| 28 | *C. brevicauda* | IAvH-M-3349 | IAvH-M |
| 29 | *C. brevicauda* | IAvH-M-7177 | IAvH-M |
| 30 | *C. brevicauda* | IAvH-M-3348 | IAvH-M |
| 31 | *C. brevicauda* | IAvH-M-3346 | IAvH-M |
| 32 | *C. brevicauda* | IAvH-M-3352 | IAvH-M |
| 33 | *C. brevicauda* | IAvH-M-2360 | IAvH-M |
| 34 | *C. brevicauda* | IAvH-M-3344 | IAvH-M |
| 35 | *C. brevicauda* | IAvH-M-2033 | IAvH-M |
| 36 | *C. brevicauda* | IAvH-M-3342 | IAvH-M |
| 37 | *C. brevicauda* | IAvH-M-2365 | IAvH-M |
| 38 | *C. brevicauda* | IAvH-M-3353 | IAvH-M |
| 39 | *C. brevicauda* | 14658 | ICN |
| 40 | *C. brevicauda* | 15103 | ICN |
| 41 | *C. brevicauda* | 15102 | ICN |
| 42 | *C. brevicauda* | 10901 | ICN |
| 43 | *C. brevicauda* | 10900 | ICN |
| 44 | *C. brevicauda* | 14657 | ICN |
| 45 | *C. brevicauda* | 8448 | ICN |
| 46 | *C. brevicauda* | 8449 | ICN |
| 47 | *C. brevicauda* | 8447 | ICN |
| 48 | *C. brevicauda* | 8445 | ICN |
| 49 | *C. brevicauda* | 17132 | ICN |
| 50 | *C. brevicauda* | 17133 | ICN |
| 51 | *C. brevicauda* | 16296 | ICN |
| 52 | *C. brevicauda* | 16295 | ICN |
| 53 | *C. brevicauda* | 9854 | ICN |
| 54 | *C. brevicauda* | 8334 | ICN |
| 55 | *C. brevicauda* | 8335 | ICN |
| 56 | *C. brevicauda* | 8326 | ICN |
| 57 | *C. brevicauda* | 8325 | ICN |
| 58 | *C. brevicauda* | 14860 | ICN |
| 59 | *C. brevicauda* | 9853 | ICN |
| 60 | *C. brevicauda* | 14859 | ICN |
| 61 | *C. brevicauda* | 9852 | ICN |
| 62 | *C. brevicauda* | 18072 | ICN |
| 63 | *C. brevicauda* | 18074 | ICN |
| 64 | *C. brevicauda* | 19908 | ICN |
| 65 | *C. brevicauda* | 19907 | ICN |
| 66 | *C. brevicauda* | 14659 | ICN |
| 67 | *C. brevicauda* | 10817 | ICN |
| 68 | *C. brevicauda* | 10821 | ICN |
| 69 | *C. brevicauda* | 14659 | ICN |
| 70 | *C. brevicauda* | 16905 | ICN |
| 71 | *C. brevicauda* | 16906 | ICN |
| 72 | *C. brevicauda* | 16908 | ICN |
| 73 | *C. brevicauda* | 16909 | ICN |
| 74 | *C. brevicauda* | 11173 | ICN |
| 75 | *C. brevicauda* | 14347 | ICN |
| 76 | *C. brevicauda* | 5227 | ICN |
| 77 | *C. brevicauda* | 16657 | ICN |
| 78 | *C. brevicauda* | 11471 | ICN |
| 79 | *C. brevicauda* | 11470 | ICN |
| 80 | *C. brevicauda* | 9171 | ICN |
| 81 | *C. brevicauda* | 9170 | ICN |
| 82 | *C. brevicauda* | 9181 | ICN |
| 83 | *C. brevicauda* | 9182 | ICN |
| 84 | *C. brevicauda* | 6036 | ICN |
| 85 | *C. brevicauda* | 6044 | ICN |
| 86 | *C. brevicauda* | 6043 | ICN |
| 87 | *C. brevicauda* | 16656 | ICN |
| 88 | *C. brevicauda* | 6035 | ICN |
| 89 | *C. brevicauda* | 15345 | ICN |
| 90 | *C. brevicauda* | 15348 | ICN |
| 91 | *C. brevicauda* | 8954 | ICN |
| 92 | *C. brevicauda* | 8952 | ICN |
| 93 | *C. brevicauda* | 7619 | ICN |
| 94 | *C. brevicauda* | 7618 | ICN |
| 95 | *C. brevicauda* | 13045 | ICN |
| 96 | *C. brevicauda* | 13049 | ICN |
| 97 | *C. brevicauda* | 17945 | ICN |
| 98 | *C. brevicauda* | 17939 | ICN |
| 99 | *C. brevicauda* | 11213 | ICN |
| 100 | *C. brevicauda* | 18065 | ICN |
| 101 | *C. brevicauda* | 18066 | ICN |
| 102 | *C. brevicauda* | 6613 | ICN |
| 103 | *C. brevicauda* | 6614 | ICN |
| 104 | *C. brevicauda* | 17886 | ICN |
| 105 | *C. brevicauda* | 17887 | ICN |
| 106 | *C. brevicauda* | 16952 | ICN |
| 107 | *C. brevicauda* | 3435 | ICN |
| 108 | *C. brevicauda* | 14346 | ICN |
| 109 | *C. castanea* | MUJ1565 | MPUJ |
| 110 | *C. castanea* | MUJ1553 | MPUJ |
| 111 | *C. castanea* | MUJ1317 | MPUJ |
| 112 | *C. castanea* | MUJ857 | MPUJ |
| 113 | *C. castanea* | MUJ683 | MPUJ |
| 114 | *C. castanea* | MUJ682 | MPUJ |
| 115 | *C. castanea* | IAvH-M-925 | IAvH-M |
| 116 | *C. castanea* | IAvH-M-3363 | IAvH-M |
| 117 | *C. castanea* | IAvH-M-4189 | IAvH-M |
| 118 | *C. castanea* | IAvH-M-2072 | IAvH-M |
| 119 | *C. castanea* | IAvH-M-5101 | IAvH-M |
| 120 | *C. castanea* | IAvH-M-5791 | IAvH-M |
| 121 | *C. castanea* | IAvH-M-4648 | IAvH-M |
| 122 | *C. castanea* | IAvH-M-2079 | IAvH-M |
| 123 | *C. castanea* | IAvH-M-4237 | IAvH-M |
| 124 | *C. castanea* | IAvH-M-4647 | IAvH-M |
| 125 | *C. castanea* | IAvH-M-506 | IAvH-M |
| 126 | *C. castanea* | IAvH-M-5820 | IAvH-M |
| 127 | *C. castanea* | IAvH-M-972 | IAvH-M |
| 128 | *C. castanea* | IAvH-M-5091 | IAvH-M |
| 129 | *C. castanea* | IAvH-M-964 | IAvH-M |
| 130 | *C. castanea* | IAvH-M-5092 | IAvH-M |
| 131 | *C. castanea* | IAvH-M-5093 | IAvH-M |
| 132 | *C. castanea* | IAvH-M-926 | IAvH-M |
| 133 | *C. castanea* | IAvH-M-3364 | IAvH-M |
| 134 | *C. castanea* | IAvH-M-2074 | IAvH-M |
| 135 | *C. castanea* | IAvH-M-2375 | IAvH-M |
| 136 | *C. castanea* | IAvH-M-2376 | IAvH-M |
| 137 | *C. castanea* | IAvH-M-2369 | IAvH-M |
| 138 | *C. castanea* | 12495 | ICN |
| 139 | *C. castanea* | 10813 | ICN |
| 140 | *C. castanea* | 14808 | ICN |
| 141 | *C. castanea* | 12496 | ICN |
| 142 | *C. castanea* | 10812 | ICN |
| 143 | *C. castanea* | 14809 | ICN |
| 144 | *C. castanea* | 16303 | ICN |
| 145 | *C. castanea* | 18981 | ICN |
| 146 | *C. castanea* | 18982 | ICN |
| 147 | *C. castanea* | 18710 | ICN |
| 148 | *C. castanea* | 18684 | ICN |
| 149 | *C. castanea* | 11296 | ICN |
| 150 | *C. castanea* | 12649 | ICN |
| 151 | *C. castanea* | 12648 | ICN |
| 152 | *C. castanea* | 11295 | ICN |
| 153 | *C. castanea* | 15862 | ICN |
| 154 | *C. castanea* | 15861 | ICN |
| 155 | *C. castanea* | 9802 | ICN |
| 156 | *C. castanea* | 9801 | ICN |
| 157 | *C. castanea* | 18220 | ICN |
| 158 | *C. castanea* | 6730 | ICN |
| 159 | *C. castanea* | 6713 | ICN |
| 160 | *C. castanea* | 6712 | ICN |
| 161 | *C. castanea* | 10852 | ICN |
| 162 | *C. castanea* | 6711 | ICN |
| 163 | *C. castanea* | 9535 | ICN |
| 164 | *C. castanea* | 10853 | ICN |
| 165 | *C. castanea* | 11750 | ICN |
| 166 | *C. castanea* | 11749 | ICN |
| 167 | *C. castanea* | 9536 | ICN |
| 168 | *C. castanea* | 12305 | ICN |
| 169 | *C. castanea* | 12304 | ICN |
| 170 | *C. castanea* | 12282 | ICN |
| 171 | *C. castanea* | 18750 | ICN |
| 172 | *C. castanea* | 482 | ICN |
| 173 | *C. castanea* | 12283 | ICN |
| 174 | *C. castanea* | 18747 | ICN |
| 175 | *C. castanea* | 686 | ICN |
| 176 | *C. castanea* | 13060 | ICN |
| 177 | *C. castanea* | 13059 | ICN |
| 178 | *C. castanea* | 8912 | ICN |
| 179 | *C. castanea* | 8911 | ICN |
| 180 | *C. castanea* | 10208 | ICN |
| 181 | *C. castanea* | 9200 | ICN |
| 182 | *C. castanea* | 12212 | ICN |
| 183 | *C. castanea* | 12213 | ICN |
| 184 | *C. castanea* | 14407 | ICN |
| 185 | *C. castanea* | 14406 | ICN |
| 186 | *C. castanea* | 17947 | ICN |
| 187 | *C. castanea* | 17948 | ICN |
| 188 | *C. castanea* | 856 | MPUJ |
| 189 | *C. castanea* | 685 | MPUJ |
| 190 | *C. castanea* | 914 | MPUJ |
| 191 | *C. perspicillata* | MUJ276 | MPUJ |
| 192 | *C. perspicillata* | MUJ1549 | MPUJ |
| 193 | *C. perspicillata* | MUJ263 | MPUJ |
| 194 | *C. perspicillata* | MUJ1624 | MPUJ |
| 195 | *C. perspicillata* | MUJ1081 | MPUJ |
| 196 | *C. perspicillata* | MUJ1530 | MPUJ |
| 197 | *C. perspicillata* | MUJ1474 | MPUJ |
| 198 | *C. perspicillata* | MUJ1256 | MPUJ |
| 199 | *C. perspicillata* | MUA11608 | CTUA |
| 200 | *C. perspicillata* | MUA11278 | CTUA |
| 201 | *C. perspicillata* | MUA10230 | CTUA |
| 202 | *C. perspicillata* | MUA10648 | CTUA |
| 203 | *C. perspicillata* | MUA10197 | CTUA |
| 204 | *C. perspicillata* | MUA10553 | CTUA |
| 205 | *C. perspicillata* | MUA11281 | CTUA |
| 206 | *C. perspicillata* | MUA10198 | CTUA |
| 207 | *C. perspicillata* | MUA11400 | CTUA |
| 208 | *C. perspicillata* | MUA10539 | CTUA |
| 209 | *C. perspicillata* | MUJ862 | MPUJ |
| 210 | *C. perspicillata* | MUJ1584 | MPUJ |
| 211 | *C. perspicillata* | MUJ1582 | MPUJ |
| 212 | *C. perspicillata* | IAvH-M-5819 | IAvH-M |
| 213 | *C. perspicillata* | IAvH-M-2380 | IAvH-M |
| 214 | *C. perspicillata* | IAvH-M-2379 | IAvH-M |
| 215 | *C. perspicillata* | IAvH-M-3377 | IAvH-M |
| 216 | *C. perspicillata* | IAvH-M-2092 | IAvH-M |
| 217 | *C. perspicillata* | IAvH-M-5800 | IAvH-M |
| 218 | *C. perspicillata* | IAvH-M-2084 | IAvH-M |
| 219 | *C. perspicillata* | IAvH-M-3382 | IAvH-M |
| 220 | *C. perspicillata* | IAvH-M-5085 | IAvH-M |
| 221 | *C. perspicillata* | IAvH-M-5083 | IAvH-M |
| 222 | *C. perspicillata* | IAvH-M-6854 | IAvH-M |
| 223 | *C. perspicillata* | IAvH-M-7107 | IAvH-M |
| 224 | *C. perspicillata* | IAvH-M-7110 | IAvH-M |
| 225 | *C. perspicillata* | IAvH-M-6775 | IAvH-M |
| 226 | *C. perspicillata* | IAvH-M-7045 | IAvH-M |
| 227 | *C. perspicillata* | IAvH-M-6724 | IAvH-M |
| 228 | *C. perspicillata* | IAvH-M-7013 | IAvH-M |
| 229 | *C. perspicillata* | IAvH-M-4570 | IAvH-M |
| 230 | *C. perspicillata* | IAvH-M-4577 | IAvH-M |
| 231 | *C. perspicillata* | IAvH-M-4603 | IAvH-M |
| 232 | *C. perspicillata* | IAvH-M-4608 | IAvH-M |
| 233 | *C. perspicillata* | IAvH-M-4586 | IAvH-M |
| 234 | *C. perspicillata* | IAvH-M-4587 | IAvH-M |
| 235 | *C. perspicillata* | IAvH-M-6855 | IAvH-M |
| 236 | *C. perspicillata* | IAvH-M-5102 | IAvH-M |
| 237 | *C. perspicillata* | IAvH-M-5100 | IAvH-M |
| 238 | *C. perspicillata* | IAvH-M-3537 | IAvH-M |
| 239 | *C. perspicillata* | IAvH-M-4232 | IAvH-M |
| 240 | *C. perspicillata* | IAvH-M-4228 | IAvH-M |
| 241 | *C. perspicillata* | IAvH-M-4225 | IAvH-M |
| 242 | *C. perspicillata* | IAvH-M-4227 | IAvH-M |
| 243 | *C. perspicillata* | 16641 | ICN |
| 244 | *C. perspicillata* | 16639 | ICN |
| 245 | *C. perspicillata* | 11734 | ICN |
| 246 | *C. perspicillata* | 11733 | ICN |
| 247 | *C. perspicillata* | 900 | ICN |
| 248 | *C. perspicillata* | 16018 | ICN |
| 249 | *C. perspicillata* | 14816 | ICN |
| 250 | *C. perspicillata* | 16019 | ICN |
| 251 | *C. perspicillata* | 11301 | ICN |
| 252 | *C. perspicillata* | 11300 | ICN |
| 253 | *C. perspicillata* | 8339 | ICN |
| 254 | *C. perspicillata* | 6000 | ICN |
| 255 | *C. perspicillata* | 19281 | ICN |
| 256 | *C. perspicillata* | 885 | ICN |
| 257 | *C. perspicillata* | 19284 | ICN |
| 258 | *C. perspicillata* | 8330 | ICN |
| 259 | *C. perspicillata* | 10959 | ICN |
| 260 | *C. perspicillata* | 5999 | ICN |
| 261 | *C. perspicillata* | 9705 | ICN |
| 262 | *C. perspicillata* | 9488 | ICN |
| 263 | *C. perspicillata* | 9704 | ICN |
| 264 | *C. perspicillata* | 9487 | ICN |
| 265 | *C. perspicillata* | 15001 | ICN |
| 266 | *C. perspicillata* | 15002 | ICN |
| 267 | *C. perspicillata* | 6716 | ICN |
| 268 | *C. perspicillata* | 6715 | ICN |
| 269 | *C. perspicillata* | 14960 | ICN |
| 270 | *C. perspicillata* | 14959 | ICN |
| 271 | *C. perspicillata* | 14532 | ICN |
| 272 | *C. perspicillata* | 14523 | ICN |
| 273 | *C. perspicillata* | 11175 | ICN |
| 274 | *C. perspicillata* | 11174 | ICN |
| 275 | *C. perspicillata* | 1385 | MPUJ |
| 276 | *C. perspicillata* | 680 | MPUJ |
| 277 | *C. perspicillata* | 1228 | MPUJ |
| 278 | *C. perspicillata* | 681 | MPUJ |
| 279 | *C. perspicillata* | 796 | MPUJ |
| 280 | *C. perspicillata* | 1295 | MPUJ |
| 281 | *C. perspicillata* | 1387 | MPUJ |
| 282 | *C. perspicillata* | 679 | MPUJ |
| 283 | *C. perspicillata* | 1258 | MPUJ |
| 284 | *C. perspicillata* | 745 | MPUJ |
| 285 | *C. perspicillata* | 1409 | MPUJ |
| 286 | *C. perspicillata* | 864 | MPUJ |
