# Supplementary material for: Cranial and mandibular shape variation in the genus Carollia (Mammalia: Chiroptera) from Colombia: biogeographic patterns and morphological modularity
Source: PeerJ. 2015 Aug 13;3:e1197. doi: 10.7717/peerj.1197 (PMC4581772; doi:10.7717/peerj.1197)
Supplement: Table S2 — Description of landmarks used to evaluate cranium and mandible shape variation. [file peerj-03-1197-s002.docx]

| Structure | Landmark | Description |
| --- | --- | --- |
| Cranium | 1 | Anterior margin of incisive along midsagittal line |
| Cranium | 2 | Anterior margin of the vomer |
| Cranium | 3 | Anterior margin of the sphenoid |
| Cranium | 4 | Anterior margin of the foramen magnum |
| Cranium | 5 | Posterior margin of the foramen magnum |
| Cranium | 6 | Occipitomastoid suture |
| Cranium | 7 | Ventral most extension of the mastoid process |
| Cranium | 8 | Dorsal border of the external auditory meatus |
| Cranium | 9 | Posterior extension of the temporal on the zigomatic arch |
| Cranium | 10 | Anterior extension of the squamosal on the zygomatic arch |
| Cranium | 11 | Posterolateral border of the vomer |
| Cranium | 12 | Anterolateral border of the vomer |
| Cranium | 13 | Posterior border of the last molar alveolus |
| Cranium | 14 | Intersection of the last premolar and first molar alveoli |
| Cranium | 15 | Posterior border of canine alveolus |
| Mandible | 1 | Anterior border of canine alveolus |
| Mandible | 2 | Posterior border of canine alveolus |
| Mandible | 3 | Posterior border of the last molar alveolus |
| Mandible | 4 | Anterolateral margin of the ramus |
| Mandible | 5 | Lateral most extension of the coronoid process |
| Mandible | 6 | Anterolateral tip of condilar process |
| Mandible | 7 | Anterolateral tip of angular process |
| Mandible | 8 | Posterolateral border of angular process |
| Mandible | 9 | Posterior-most point on the baseline perpendicular to the landmark 5 |
| Mandible | 10 | Anterior-most point on the baseline perpendicular to the landmark 3 |
| Mandible | 11 | Dorsal-most point on the border of the mandible |
| Mandible | 12 | Anterior lower border of the mandible |

**Table A2.** Description of landmarks usted to evaluate cranium and mandible shape variation
